# Renal function, calcium regulation, and time to hospitalization of patients with chronic kidney disease

**DOI:** 10.1186/1471-2369-14-154

**Published:** 2013-07-18

**Authors:** Mojgan Golzy, Russell W Bessette, Randy L Carter

**Affiliations:** 1 Department of Biostatistics and Population Health Observatory, State University of New York at Buffalo, Buffalo, NY 14214-3000, USA; 2 Abell Administration Center, University of Louisville, 323 East Chestnut St., Louisville, Kentucky 40202, USA

**Keywords:** Quality of care metric, Risk assessment, Prediction, Censored Weibull regression, Path modeling, Direct and indirect effects

## Abstract

**Background:**

Chronic kidney disease is associated with disruption of the endocrine system that distorts the balance between calcitriol, calcium, phosphate and parathyroid hormone in the calcium regulation system. This can lead to calcification of the arterial tree and increased risk of cardiovascular disease and death. In this study we develop a health metric, based on biomarkers involved in the calcium regulation system, for use in identifying patients at high risk for future high-cost complications.

**Methods:**

This study is a retrospective observational study involving a secondary analysis of data from the kidney disease registry of a regional managed care organization. Chronic kidney disease patients in the registry from November 2007 through November 2011 with a complete set of observations of estimated glomerular filtration rate, calcitriol, albumin, free calcium, phosphate, and parathyroid hormone were included in the study (n = 284). Weibull regression model was used to identify the most significant lab tests in predicting “waiting time to hospitalization”. A multivariate linear path model was then constructed to investigate direct and indirect effects of the biomarkers on this outcome.

**Results:**

The results showed negative significant direct effects of phosphate and parathyroid hormone on “waiting time to hospitalization”. Base on this result, the risk of hospitalization increases 16.8% for each 0.55 mg/dl increase in phosphate level and 13.5% for each 0.467 increase in the natural logarithm of parathyroid hormone. Positive indirect effects of calcitriol surrogate (calcidiol), free calcium, albumin and estimated glomerular filtration rate were observed but were relatively small in magnitude.

**Conclusion:**

Variables involved in the calcium regulation system should be included in future efforts to develop a quality of care index for Chronic Kidney disease patients.

## Background

Kidney disease is the ninth leading cause of death in the United States [1]. Chronic kidney disease (CKD) is associated with disruption of the endocrine system that distorts the balance between calcitriol, calcium, phosphate (PO4) and parathyroid hormone (PTH) in the calcium regulation system [1-4]. This can lead to calcification of the arterial tree and increased risk of cardiovascular disease (CVD) and death [5-8]. There are different opinions on which serum enzymes or minerals reliably predict advancing illness and high cost healthcare in CKD patients [9]. Bessette and Carter [9] employed multivariable logistic regression to investigate which serum chemistry values are significantly associated with in-patient hospital costs exceeding $3,000 in any single month. Their results suggested the calcium regulation system may play an important role in the health and cost of treatment of patients with CKD. In that paper, due to the small sample size and short follow up period, the average costs and average lab results over a 13 months period were analyzed. Averaging would be a limitation in the current study given our desired to describe causal relationships. Given a longer follow up period of 4 years and a larger sample size, we now can more precisely assess the impact of kidney function and calcium homeostasis imbalances on the risk of hospitalization using longitudinal instead of averaged data.

The main purpose of this study is to develop a health metric, based on variables involved in the calcium regulation system (calcium, PO4, PTH), a kidney function indicator (eGFR), and a set of kidney function associates (calcitriol and albumin), that is related to the risk of future high-cost complications. We test the significance of estimated direct and indirect effects of these kidney function measures and serum chemistry values on waiting time to hospitalization in CKD patients with widely varying severity of disease.

The National Kidney Foundation (NKF) defines CKD as either kidney damage or a sustained kidney glomerular filtration rate (GFR) of less than 60 ml/min/1.73 *m*^2^ for 3 or more months [10]. An estimate of GFR (eGFR) can be calculated from the patient’s routine blood tests. The highest incidence rate of End-stage Renal Disease (ESRD) occurs in patients older than 65 years [11]. Age, diabetes mellitus, and hypertension are major predictors of chronic kidney disease [11].

**Figure 1:**
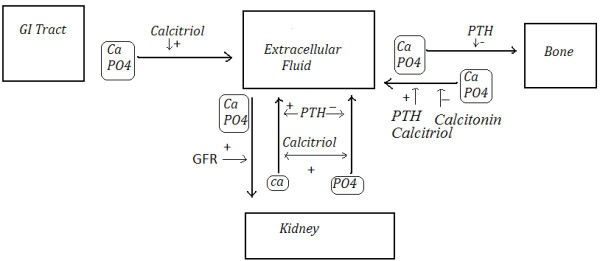
**Interaction between calcium, phosphate, PTH, and calcitriol **[12]**.**

Phosphate (PO4), calcium, activated vitamin D 1,25-dihydroxyvitamin *D*_3_ (calcitriol), parathyroid hormone (PTH) and their influence on kidney function play an important role in controlling the level of phosphate and calcium in the bloodstream. Healthy kidneys are rich with 1-alpha hydroxylase enzyme, which plays a major role in turning vitamin D into its active form, calcitriol. Calcitriol acts on vitamin D receptors, which control calcium channels and therefore is an important component of the calcium regulation system. Secondarily, it controls the absorption of phosphate and in turn regulates PTH levels. When kidneys fail, their ability to activate vitamin D is diminished. Without the activated vitamin D to facilitate calcium and phosphate absorption, PTH will increase, which results in calcium and phosphate resorption from the bone [12,13] (see Figure 1). An illustration of the interaction between calcium, phosphate, PTH, and calcitriol is presented in Figure 1. Silver et al. [3], and Palmer et al. [8] provide additional information.

## Method

### Data

The data set analyzed contains blood test results: eGFR, calcidiol, albumin, Ca ^++^, PO4, and PTH from 284 de-identified patients. The data did not include the measurement of the serum calcitriol and so we use 25hydroxyvitamin D (calcidiol) as a surrogate for serum calcitriol. There is a significant biphasic relationship between serum calcitriol and serum calcidiol. This relation is positive at normal calcidiol levels, because of the effect of substrate deficiency on calcitriol production, but negative at low calcidiol levels, because secondary hyperparathyroidism simulates the synthesis of calcitriol [14]. About 90% of the serum calcidiol measurements, in our data set, were in normal range. So, it is reasonable to used the strongly correlated calcitriol as a surrogate in our study. Therefore, calcidiol effects found in our analyses can be interpreted as calcitriol effects.

The data set were drawn from the kidney disease registry of a managed care organization (MCO) during the 4 year period from Nov. 2007 through Nov. 2011. The data set also included age, gender, service date and a complete financial profile for all medical claims that were paid for these patients over that same time period. Service date is a date that medical services were provided. A service constitutes lab tests, procedures, hospital services, physician services, office visits, or other services for which claims were submitted. Charges for services were matched with corresponding date of service. This data was obtained after review of the study protocol by the university at Buffalo’s Health Sciences Institutional Review Board (HSIRB) and permission from the regional MCO.

The registry contained records of 14,264 patients who were treated for kidney disease during this time period. 5,799 of those had confirmed CKD (as defined by eGFR calculated using the MDRD equation of less than 60 ml/min/1.73 *m*^2^ for 3 or more months.) 284 of those had complete observation (see subsequent paragraphs). We compared the 284 selected patients (study group) to the remaining 5,515 CKD patients to check the representativeness of the sample analyzed. For each individual, we calculated the average lab tests over the entire time period and then used PROC TTEST in SAS software. Table 1 gives the summary of the results as the number of observations, mean, and standard deviation for each group and the pooled p-value for mean differences for each variable. Considering the significant level *α*=0.05/7=0.007, using the Bonferroni correction, the means are not significantly different. Thus, we have no significant evidence that the sample of 284 patients analyzed in this study are not representative of all CKD patients in the registry.

**Table 1:** Comparison of the study group and the remaining other CKD patients

**Variable**	**Group**	**N**	**Mean**	**Unit**	**Standard**	**p-value**
					**deviation**	
Age	Study group^∗^	284	67.9	Years	12.9	0.09
	Other CKD pat.^∗∗^	5367	69.4		14.4	
eGFR	Study group	284	32.77	mL/min/1.73m	11.9	0.04
	Other CKD pat.	5515	30.6		17.48	
Albumin	Study group	284	4.13	gm/dl	0.29	0.02
	Other CKD pat.	4946	4.08		0.4	
Calcidiol	Study group	284	26.6	ng/ml	13.7	0.6
	Other CKD pat.	1492	27.01		13.54	
PTH	Study group	284	96.3	pg/ml	106.6	0.6
	Other CKD pat.	819	93.27		113.7	
PO4	Study group	284	3.61	mg/dl	0.60	0.3
	Other CKD pat.	1296	3.66		0.68	
*Ca*^++^	Study group	284	5.25	mg/dl	0.45	0.3
	Other CKD pat.	4535	5.22		0.41	

The number of observations, mean and standard deviation for each group and the pooled p-value for mean differences for each variable. (^∗^) Study group is the confirmed CKD patients in the registry with use-able complete observations. (^∗∗^) The remaining confirmed CKD patients in the registry (as defined by at least one eGFR calculated and max{eGFR} ≤ 60 ml/min/1.73 *m*^2^).

In this data set, a “hospitalization” is said to have occurred when a month of charges exceeded $3,000 and those charges were confirmed to be for hospital services. We defined the date of hospitalization to be the service date with maximum cost in a month with monthly cost greater than $3,000. For each individual, we determined the dates of all hospitalizations, if any.

### Definition of variables

It is generally agreed that the ionized (or free) calcium is the form that is biologically active. Because of this, free calcium (Ca^++^) is a more useful index than total calcium and provides a better indication of calcium status [15-17]. Ionized calcium can be measured directly with the use of calcium-specific electrodes. If ionized calcium cannot be measured, however, certain approximations can be utilized to distinguish the protein bound calcium from the ionized fraction calcium. We adopt the following formula from [15] to calculate free calcium.

$$\begin{array}{lll} & \%\;\text{protein-bound calcium}\approx 0.8\;\text{albumin}(g/l)\; & \;\\ & \;+0.2\;\text{globulin}\;(g/l)+3\; & \;\\ & \text{Free calcium}\;({\text{ionzed calcium Ca}}^{++})\approx \;\text{total calcium}\; & \;\\ & \;-\text{protein bound to calcium}\; & \; \end{array}$$

For patients with at least one hospitalization, we searched the hospitalization intervals, from the first to the last, for a complete set of observation of lab tests of interest. Hospitalization intervals are; 3 weeks after the first service date until 3 weeks before the first hospitalization, 3 weeks after one hospitalization until 3 weeks before the next hospitalization, and 3 weeks after the last hospitalization until the last service date. We did not include lab tests taken within 3 weeks before a hospitalization due to the fact that these tests could be taken in preparation for that hospitalization, which would make for an artificially short waiting time to hospitalization. We also excluded test taken within 3 weeks after the previous hospitalization due to the fact that these tests may reflect a causal association of last hospitalization event on test scores which is not direction of causation we want to study.

To search for a complete set of lab tests in an interval, we selected the first set of observed lab test scores in that interval. Since PO4 comes last in our causal ordering we selected an observation of PO4 which a complete set of other lab tests was observed before or simultaneously with the selected observation. If the first interval did not contain a complete set of lab scores then we moved to the next interval and continued in this fashion until an interval with a complete set of lab scores was observed. If a complete set was not observed in at least one interval then the patient was not included in the analysis data set. After completing this process for each patient, 284 patients remained in the data set to be analyzed.

**Figure 2:**
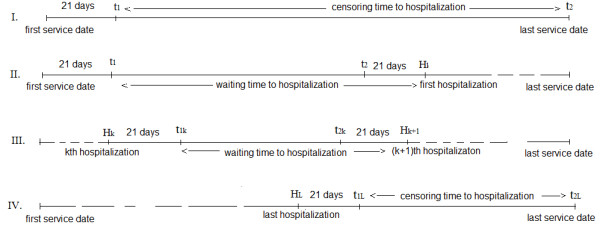
**Calculation of waiting time to hospitalization in four situations. I.** Patients with no hospitalization with a complete set of observations after *t*_1_, where *t*_1_ is 21 days after the first service date. **II.** Patients with hospitalization with a complete set of observations in the first hospitalization interval [ *t*_1_,*t*_2_], where *t*_1_ is 21 days after the first service date and *t*_2_ is 21 days before the first hospitalization. **III.** Patients with hospitalization who had their first complete set of observation in the *k*^*th*^hospitalization interval [ *t*_1*k*_,*t*_2*k*_], where *t*_1*k*_is 21 days after the *k*^*th*^hospitalization and *t*_2*k*_is 21 days before the (*k*+1)^*th*^hospitalization. **IV.** Patients with hospitalization who had their first complete set of observation in the last hospitalization interval [ *t*_1*l*_,*t*_2*l*_], where *t*_1*l*_is 21 days after the last hospitalization and *t*_2*l*_is the last service date.

To define the outcome variable, waiting time to hospitalization (WTH), we considered the hospitalization interval in which a complete set of lab tests was observed and then defined the waiting time to hospitalization by the time from the left of that interval until the next hospitalization. For individuals with no hospitalization, we selected the first lab tests in the time interval from 3 weeks after first service date until the last service date. For these individuals with no hospitalization, waiting time to hospitalization is censored at C, where C is the time from 3 weeks after the first service date until the last service date. Also for individuals with at least one hospitalization and with a complete set of lab observations only after the last hospitalization, waiting time to hospitalization is also censored at C, where C is the time from 3 weeks after the last hospitalization until the last service date. (see Figure 2)

Normal probability plots were used to assess the normality of each variable (in a statistical sense). If a variable was non-normal a normalizing transformation was applied. We used the natural log transformation of the non-normal variable PTH to normalize it. No transformation was required for other lab variables because they appeared to be normally distributed, based on normal probability plot. The normal limits for lab test scores studied are given in Table 2. Normal limits for ln(PTH) were calculated by similarly transforming the upper and lower normal limits of PTH.

**Table 2:** The normal limits for the lab test scores and transformed lab test scores

**Variable**	**Normal limits**	**Unit**
eGFR	Male (100, 140), Female (85, 115)	mL/min/1.73m2
Albumin	(3.6, 5)	gm/dl
Calcidiol	(9.7, 41.7)	ng/ml
PTH	(10, 65)	pg/ml
ln(PTH)	(2.3, 4.17)	
PO4	(2.1, 4.3)	mg/dl
*Ca*^++^	(4.5, 5.6)	mg/dl

Using the normal limits in Table 2, we standardized each ln(PTH) and each variable that did not require transformation. The average of the corresponding normal limits was taken as the mean, and the range of the normal limits divided by 4 as the standard deviation in the standardization calculations. Let z-eGFR, z-albumin, z-calcidiol, z-PTH, z-PO4 and *z-Ca*^++^denote the z-scores of the corresponding variables (transformed variable in the case of PTH).

### Statistical methods

We first used a censored regression model [18,19] to estimate the effect of each kidney function and each calcium homeostasis covariate while controlling for other covariates on the mean of the natural log of waiting time to hospitalization. The other variables in causal ordering given in the next section are modeled as function of the previous variables in that ordering, using multiple regression analysis.

In the censored regression model *ln*(*Y*)=*μ*+*γ*^′^*Z*+*σ*.*ε* with Weibull distribution for Y, *σ* is the scale parameter and *ε* is the error term. *Z* is vector of covariates and *γ* is the vector of regression coefficients, which represent the associations of each covariate with ln(Y). The values of a Weibull random variable range over the interval (0,*∞*) and WTH >21 is garanteed given its definition in section ‘Definition of variables’, WTH is 21 days greater than the length of the hospitalization interval. Therefore, we used the transformation Y = WTH-21, which ranges from zero up, to better fit the Weibull model. Plot of the log of the negative log of the estimated survival function against log time (LLS plot) provided a visual check of the appropriateness of the Weibull model for Y.

We chose the Weibull model for the following reasons: 

• empirical evidence that it is reasonable; (We graphically assessed whether or not the data set follows Weibull distribution by checking the Weibull probability plot,using proc lifereg in sas.)

• the ability to calculate MSE and R-square type statistics that are analogous to those calculated for multivariate linear regression models;

• the greater efficiency one can expect to achieve when using a fully parametric model versus the nonparametric Cox model;

• and, because if fits when the parametric model assumptions are satisfied a linear mean function and, thus is a convenient choice for multivariate linear path modeling. (Multivariate linear path models involve linear mean function specifications.)

The Weibull model also has a proportional hazards interpretation. We used residuals to investigate the proportional hazards assumption, using martingale and deviance residuals for the Cox proportional hazards regression analysis. There is no indication of a lack of fit of the model to individual observations. We also created plots of the cumulative martingale residuals against the values of the each covariate and computed the p-value of a Kolmogorov-type supremum tests based on a sample of 1,000 simulated residual patterns. There was no evidence to reject the proportional hazard assumption. With the Cox proportional hazards model, the regression coefficients correspond directly to the log of hazard ratios. The hazard ratio corresponding to the *k*^*th*^variable for a Weibull model is given by *exp*(−*β*_*k*_/*σ*) where *β*_*k*_is the corresponding regression coefficient for the *k*^*th*^variable and *σ* is the scale parameter.

#### Multivariate path analysis

Path analysis is a statistical technique used to examine direct and indirect relationships among a set of causally ordered variables. Such relationships, if linear, can be described in a system of linear equations with random variables of interest and unknown parameters. Path analysis was first developed by Sewall Wright in the 1930s for use in phylogenetic studies [20,21]. Classical path analysis assumes a complete casual ordering of variables with unidirectional relationships (i.e., no feedback loops.) Path analysis allows us decompose total effects of upstream variables on subsequent variables in the causal ordering into direct effects and indirect effects. While classical methods require a complete causal ordering of variables, Multivariate Linear Path Model (MLPM) allows a relaxation of this assumption, requiring only a causal ordering of sets of variables. For details we refer to Pak [22,23].

In linear path analysis direct effects, which are the corresponding regression coefficients, and indirect effects; i.e., those acting through an intermediate variable W, in the pathway *X*→*W*→*Z*, can be examined. Indirect effects can be calculated by multiplying the corresponding direct effects. For example, indirect effect of X on Z in the pathway *X*→*W*→*Z* is product of direct effect of X on W and direct effect of W on Z. The p-value for testing the indirect effects can be found using the Intersection Union Test (IUT) [24]. Total effects are defined as the sum of direct and all possible indirect effects.

In this study not all of the variables of interest can be causally ordered in a unidirectional fashion. Sets of variables, however, can be ordered. Thus, we use multivariate linear path modeling, which requires only partial ordering; i.e., an ordering of sets of variables. The models then involve multivariate regression models in place of the univariate regression models associated with the classical path analysis. In the multivariate setting causal ordering is not necessary within each set of variables and the errors in the model for those variables are not required to be independent. In this application we assume the following causal ordering.

##### The causal ordering in the multivarite path model for calcium regulation

$$\begin{array}{lll} \left[\text{age}\right] & \to \left[\begin{array}{l} \text{z-eGFR}\\ \text{z-calcidiol}\;\begin{array}{l} (\text{calcitriol}\\ \;\text{surogate}) \end{array}\\ \text{z-albumina} \end{array},\;\right]\;\to \left[{\text{z-Ca}}^{++}\right]\; & \;\\ & \to \left[\text{z-PTH}\right]\to \left[\text{z-PO4}\right]\to \left[\text{ln(Y)}\right]\; & \; \end{array}$$

Considering age as the exogenous variable and ln(Y) as the endpoint endogenous variable, we assume the following casual ordering of the variables, illustrated above. Note that we do not assume any causal ordering between z-eGFR, z-albumin and z-calcidiol, thus making our problem one that is conducive to MLPM.

The assumption of casual ordering of variables, illustrated above, is based on the review of the literature presented above. Low eGFR, low calcitriol and low albumin can be manifestations of underlying kidney damage. Calcitriol has effects on calcium levels in the bloodstream. Low calcitriol inhibits absorption of calcium more than phosphate from the intestines, while resorption from the bone is equal, thus contributing to high serum phosphate levels. Low calcium induces high PTH, which causes calcium and phosphate resorption from bone. This bone resoption raises calcium to its proper level while raising phosphate above its desired level.

Estimation of the multivariate path model parameters is achieved by the ordinary least square method [24,25] applied separately to each model (univariate or multivariate, as appropriate) for all variables except ln(Y) = ln(WTH-21). For ln(Y) and associated censored regression model, ordinary least square estimation of the parameters would produce biased estimators. So, we used PROC LIFEREG, in SAS software [19], which estimates the parameters using maximum likelihood estimation for regression of the lab tests on ln(Y), assuming a Weibull distribution for Y.

For the measures of goodness of fit, in the simple linear regression models, we used the squared coefficient of multiple determination, *R*^2^, to measure the predictivity of the model outcome variable in terms of explained variability of the dependent variable [25]. We note that for the censored regression model, *R*^2^can not be calculated due to censoring. So for the measure of explained variation by this model, we used the Kent and O’Quigley [26] product-moment correlation coefficient $R_{\text{PM}}^{2}$ to measure the strength of relationship of ln(Y) with the covariates, in total. For the Censored regression model given by *ln*(*Y*)=*μ*+*γ*^′^*Z*+*σ*.*ε*, we have

$$R_{\text{PM}}^{2}=\frac{\text{Var}(\gamma^{\prime }Z)}{\text{Var}(\gamma^{\prime }Z)+\sigma^{2}\sigma_{\varepsilon }^{2}}$$

where *σ* is the scale parameter and $\sigma_{\varepsilon }^{2}=\pi^{2}/6\approx 1.645$ is the variance of error term in the Weibull model. This quantity was estimated by

$${\hat{R}}_{\text{PM}}^{2}=\frac{\text{Sample variance}({\hat{\gamma }}^{\prime }Z)}{\text{Sample variance}({\hat{\gamma }}^{\prime }Z)+{\hat{\sigma }}^{2}\sigma_{\varepsilon }^{2}},$$

where $\hat{\gamma }$ and $\hat{\sigma }$ are the MLE’s of *γ* and *σ* from the Weibull regression analysis.

## Results

Statistic calculations were performed using the SAS software. For the sample of 284 patients, the mean age was 66.3 years with standard deviation 12.8 (range = (21, 90) years). There were 34 patients in stage 5 (kidney failure with eGFR ≤15), 85 patients in stage 4 (kidney damage with severely low eGFR 16-29), and 165 patients in stage 3 (kidney damage with moderately low eGFR 30-60).

There were 70 individual with no hospitalization out of the 284 patients in our study and, hence, had censored observation. From the 214 individuals with hospitalization only 137 patients had a complete set of observed lab tests prior to the next hospitalization and 77 patients had a complete set of observed lab tests only after the last hospitalization. We had 147 (70 + 77) right censored values out of the 284 observations.

### Results of path modeling

Table 3 gives a summary of the results of direct effects (top value in each cell) and the p-values (bottom value) for the variables in each equation in the multivariate path model. It also gives the estimates of the mean square errors (MSE) and r-squares of the regression equations.

**Table 3:** Estimates of direct effects in the multivariate path model

**Response*→***	**z-eGFR**	**z-albumin**	**z-calcidiol**	**z-Ca*++***	**z-PTH**	**z-PO4**	**ln(Y)**
**Explanatory*↓***							
Age	0.003	-0.002	**0.018**	-0.003	0.002	-0.0007	-0.0064
	0.7	0.6	0.026	0.46	0.77	0.9	0.3
z-eGFR				**0.089**	**-0.199**	**-0.206**	-0.039
				0.015	0.001	<0.0001	0.46
z-albumin				**-0.357**	**-0.557**	**-0.208**	0.131
				<0.0001	<0.0001	0.017	0.15
z-calcidiol				**0.108**	**-0.191**	-0.046	-0.0219
				0.001	0.0008	0.3	0.61
z-Ca^++^					**-0.369**	-0.045	-0.054
					0.0002	0.57	0.54
z-PTH						0.0047	**-0.102**
						0.91	0.039
z-PO4							**-0.141**
							0.022
Intercept	**-8.87**	-0.35	-0.96	**2.34**	0.57	-1.03	**7.7**
	<0.0001	0.23	0.08	<0.0001	0.45	0.08	<0.0001
*MSE*	2.6	0.93	3.15	0.96	2.56	1.54	1.27^∗^
*R*^2^	0.0005	0.0006	0.017	0.137	0.23	0.12	0.09^∗∗^

Estimates of direct effects (top value in each cell), and the p-values (bottom value) for the variables in each model, estimates of MSE and r-squares are given. The significant values are shown in bold. (^∗^) The value of ${\hat{\sigma }}^{2}\sigma_{\varepsilon }^{2}$ is given instead of MSE. (^∗∗^) The value of ${\hat{R}}_{\text{PM}}^{2}$ is given for the explained variation measure instead of r-square.

In Table 3, each column gives the estimated equation for the variable named at the top of the table. The top value in each cell is the direct effect (regression coefficient) and the bottom value is the corresponding p-value. For example, the fourth column gives the estimated equation for z-Ca^++^, which is

$$\begin{array}{lll} \text{zC}\hat{a^{++}} & =2.34-0.003\;\text{Age}\;+0.089\;\text{zeGFR}\; & \;\\ & \;-0.357\;\text{zAlbumin}+0.108\;\text{zCalcidiol}\; & \; \end{array}$$

We note that the error term in the censored regression model for ln(Y), Y = WTH-21, is *σε* where *σ* is the scale parameter of the Weibull distribution, with estimated value $\hat{\sigma }=0.88$ from the result, and *ε* is an error with variance $\sigma_{\varepsilon }^{2}=\pi^{2}/6\approx 1.645$. Thus, the estimate of the conditional variance of the ln(Y) given covariates in the model, which is ${\hat{\sigma }}^{2}\sigma_{\varepsilon }^{2}\approx$ 1.27, is given in Table 3 instead of MSE. For this censored regression model the Kent and O’Quigley product-moment correlation coefficient measure ${\hat{R}}_{\text{PM}}^{2}=0.09$ is given, while the usual *R*^2^is presented for each other equation.

The primary purpose of path modeling is to propose a plausible interpretation of the observed data and to describe effects in terms of significant direct and indirect effects. To find the best fitted model we used backward selection with significance level *α*=0.05 for each linear model to obtain the most parsimonious model that includes only significant effects.

Table 4 gives the estimates of direct effects and the p-values for the variables that remained in the parsimonious model. It also gives the estimates of mean square errors and r-squares for the models for upstream variables in the causal ordering.

**Table 4:** Estimates of direct effects in Parsimonious model

**Response var.*→***	**z-eGFR**	**z-albumin**	**z-calcidiol**	**z-Ca*++***	**z-PTH**	**z-PO4**	**ln(Y)**	**Total effects**
**Explanatory var.*↓***								**onln(Y)**
Age			**0.017**					**0.00043**
			0.026					
z-eGFR				**0.088**	**-0.198**	**-0.213**		**0.0549**
				0.016	0.0012	<0.0001		
z-albuminl				**-0.355**	**-0.559**	**-0.214**		**0.0774**
				<0.0001	<0.0001	0.0059		
z-calcidiol				**0.105**	**-0.189**			**0.0255**
				0.0015	0.0008			
z-Ca^++^					**-0.37**			**0.0414**
					0.0002			0.011
z-PTH							**-0.112**	**-0.112**
							0.011	0.011
z-PO4							**-0.137**	**-0.137**
							0.016	0.016
Intercept	**-8.6**	**-0.49**	-0.96	**2.12**	0.715	**-1.2**	**7.49**	
	<0.001	<0.0001	0.08	<0.0001	0.2	0.0032	<0.0001	
MSE	2.6	0.96	3.15	0.95	2.5	1.54	1.27^∗^	
*R*^2^	0	0	0.017	0.14	0.23	0.11	0.078^∗∗^	

Estimates of direct effects (top value in each cell) and the p-values (bottom value) for the variables in the parsimonious model, estimates of MSE and r-squares. The calculated total effects are given in the last column. (^∗^) The value of ${\hat{\sigma }}^{2}\sigma_{\varepsilon }^{2}$ is given instead of MSE. (^∗∗^) The value of ${\hat{R}}_{\text{PM}}^{2}$ is given for the explained variation measure instead of r-square.

### Direct, indirect and total effects

**Figure 3:**
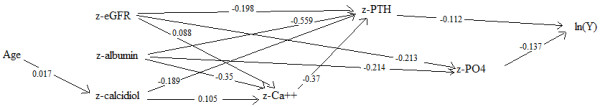
The parsimonious multivariate path model.

Figure 3 illustrates the significant associations of variables in the parsimonious multivariate path model with ln(Y). The arrows indicate single direct effects and the estimates of these direct effects are given on each arrow. The sign on each estimate of each direct effect indicates the direction of the association. Theses path coefficients measure the effect of a one standard deviation change in each original predictor variable on the response variable.

Indirect effects can be calculated by multiplying the corresponding direct effects along the indirect path from a variable to ln(Y). For example, indirect effect of X on Z in the pathway *X*→*W*→*Z* is product of direct effect of X on W and direct effect of W on Z. The p-value for testing the indirect effects can be found using the Intersection Union Test (IUT) [20,21,24]. For example, significant positive indirect effect of z-Ca^++^through z-PTH is 0.0414=(−0.37)(−0.113) with p-value = max{0.0002,0.011}=0.011. Total effects are defined as the sum of direct and all possible indirect effects.

From the result of the parsimonious path model, the direct and indirect effects of variables on ln(Y), Y = WTH-21, are as follows: 

• z-PTH and z-PO4 had significant direct effects of -0.112 (p-value = 0.011) and -0.137 (p-value = 0.016), respectively.

• z-Ca^++^had significant positive indirect effect through z-PTH of 0.0414 (p-value = 0.011.)

• z-calcidiol (calcitriol surrogate) had a significant positive indirect effect through z-PTH of 0.0212 (p-value = 0.011) and a positive indirect effect through z-Ca^++^of 0.0043. The total effect of z-Calcidiol is 0.0255.

• z-albumin had a significant positive indirect effect through z-PO4 of 0.0293, a significant indirect positive effect through z-PTH of 0.0626 and a significant indirect negative effect through z-Ca^++^of −0.0145. Thus, its total effect on ln(Y) was 0.0774.

• z-eGFR had a positive significant indirect effect through z-PO4 of 0.02918, a significant positive indirect effect through z-PTH of 0.02217 and a significant positive indirect effect through z-Ca^++^ of 0.00364. Thus, its total effect onln(Y) was 0.0549.

• Age had a significant second order indirect effects, one through z-calcidiol and then through z-PTH (0.0003598, p-value <0.05) and a third order indirect effect through z-calcidiol, z-Ca^++^and z-PTH (0.0000739, P-value <0.05) for the total effect of 0.00043. This effect is clinically insignificant.

## Discussion

From the result of the censored regression model, we identified that PTH and PO4 are the most significant predictors of high-cost hospitalization and, along with albumin, they have the largest effects (see Table 3). The prediction equation from the parsimonious model is

Predictedln(WTH−21)=7.49−0.112z PTH−0.137zPO4

The right hand side of this equation defines a calcium regulation-base health metric for CKD.

The negative significant effect of PTH on waiting time to hospitalization indicates patients with higher PTH are at higher risk of hospitalization. With Weibull model for ln(Y), the regression coefficients correspond directly to the log of hazard ratios so that a negative coefficient for a particular covariate corresponds to a higher risk of hospitalization occurring. The estimated hazard ratio corresponding to the effect of PTH is *exp*(0.112/0.88)=1.135. The hazard ratio of 1.135 indicates that the risk of hospitalization will increase 13.5% for each 1 standard deviation increase in z-PTH. A one standard deviation increase in z-PTH corresponds to an approximate 0.467 increase in the natural logarithm of PTH level (recall that the natural logarithm transform was used in defining z-PTH) or, equivalently a 60% increase in PTH.

The negative significant effect of phosphate indicates higher risk of hospitalization for patients with higher phosphate levels. A hazard ratio of 1.168 associated with this effect indicates that the risk of a hospitalization will increase 16.8% for each 1 standard deviation increase in z-PO4. A one standard deviation increase in z-PO4 is approximately 0.55 mg/dl increase in PO4 level. Thus, the risk of hospitalization is estimated to increase by 16.8% for each 0.55 mg/dl increase in PO4.

Extreme elevations of PTH levels along with hyperphosphatemia could result from an abnormal form of plasma PTH. The latter could result from an abnormal conversion of the pro-hormone to its secreted form [27].

Although, we did not find significant direct effects of eGFR, albumin, calcidiol (calcitriol surrogate) and Ca^++^on ln(Y), and hence on WTH, they are the most significant predictors of PTH and PO4. Therefore they had significant indirect effects on WTH through PTH and PO4. These effects are small, however, relative to the direct effects of PTH and PO4 on WTH.

Free calcium (Ca^++^) was not observed directly in this study. Instead a derived version [16] was used as an approximation. We repeated our analysis using total serum calcium (Ca) instead of free calcium (Ca^++^) and obtained essentially the same results. Since Ca^++^is what is regulated [15,16] by the calcium regulation system, we chose to present the analysis of Ca^++^in this paper.

Although, previous studies suggest that high serum alkaline phosphatase (ALP) levels predict mortality independent of bone metabolism parameters and liver function tests in CKD and chronic hemodialysis patients [28], we did not find any association between ALP and WTH when adding ALP in the model. Thus, we removed ALP from our model.

The current results provide a calcium regulation based health metric for identifying patients who are at high risk for future high-cost complications and that can be used as an outcome variable in assessing quality of care. Since the O’Quigley *R*^2^for the ln(Y) equation is low, however, more work is needed to identify additional lab results that are also predictive. The potential role of Fibroblast growth factor FGF-23 should be considered as candidates for addition. Fibroblast growth factor FGF-23 is a recently discovered regulator of calcium-phosphate metabolism. In CKD patients, FGF-23 levels rise in parallel with declining renal function long before a significant increase in serum phosphate concentration can be detected [29]. Unfortunately, FGF-23 was not available in our data set.

Race is known to be associated with both CKD progression and outcomes and is believed to have effects on PTH and calcium-phosphate metabolism. Our data, however, was provided without a race variable. It should be noted that the MCO calculated race specific eGFR’s and that those analyzed in this study are race adjusted. Further study should include race as a factor in addition to using it in the calculation of eGFR.

From 14,264 patients in the registry of the MCO only 5,799 had confirmed CKD. 546 of them had observed lab test scores of interest during the 4 year period of study. Out of 546 patients 284 had observed lab scores in at least one hospitalization interval. Work to develop methods of imputation in the context of longitudinal data analysis is ongoing. Once completed, the method developed will allow more complete utization of data in the kidney registry.

Additional work with data rich in lab scores is needed to identify the additional risk factors for use in predictive models to develop a health metric for assessing the quality of care of CKD patients, while adjusting for the illness severity of their case mix. Such a metric, if highly predictive of hospitalization, ESRD or death outcome could serve as the foundation of a reimbursement system that fairly rewards physicians for quality of care. The results of the current study are encouraging and justify a similar prospectively designed investigation.

## Conclusion

Variables involved in the calcium regulation system should be included in future efforts to develop a quality of care index for Chronic Kidney disease patients.

## Competing interests

The authors have no competing interests to declare.

## Authors’ contributions

RLC and RWB designed research; RLC and MG performed and analyzed the data and wrote the paper; RLC and RWB edited the manuscript. All authors read and approved the final manuscript.

## Pre-publication history

The pre-publication history for this paper can be accessed here:

http://www.biomedcentral.com/1471-2369/14/154/prepub
